# Impact of Polyimide Semi-Interpenetrating Polymer Networks on the Physicochemical Properties of Vulcanized Acrylonitrile–Butadiene Rubber

**DOI:** 10.3390/polym18182267

**Published:** 2026-09-17

**Authors:** Sunhyeong Kwon, Ji Min Kim, Geun-Su Park, Chul-Young Kim, Garrett M. Milligan, Jian Hou, Zhicheng Cai, Jun-Hyun Kim, Hyun-Ho Park

**Affiliations:** 1 Department of Chemistry, Keimyung University, Daegu 42601, Republic of Korea; gikme001@naver.com (S.K.); qudaktaosldk@naver.com (J.M.K.); 2 R&D Centers, Pyung Hwa Industrial Co., Ltd., Daegu 42982, Republic of Korea; power79@ph.co.kr (G.-S.P.); cykim@ph.co.kr (C.-Y.K.); 3 Department of Chemistry, Illinois State University, Normal, IL 61790, USA; gmillig@ilstu.edu; 4 School of Intelligent Manufacturing, Luoyang Institute of Science and Technology, Luoyang 471023, China; jhou@lit.edu.cn; 5 Department of Semiconductor System Engineering, Sejong University, Gwangjin-gu, Seoul 05006, Republic of Korea; cai1121@sejong.ac.kr

**Keywords:** acrylonitrile butadiene rubber, polyimide, interpenetrating polymer network, interconnected network, thermoset elastomer, polyamic acid

## Abstract

This work demonstrates the physicochemical property changes of composite thermoset elastomers prepared by incorporating mechanically and thermally stable polyimide (PI) into vulcanized acrylonitrile butadiene rubber (NBR) through a semi-interpenetrating polymer network (semi-IPN). Conventional NBR base materials and fillers were initially mixed with PI precursors in various forms—solid monomers, solid/liquid mixed monomers, and a liquid polyamic acid (PAA)—using a Banbury mixer, followed by processing on an open roll mill. Upon vulcanization at elevated temperature, linear PI was incorporated into the crosslinked NBR matrix to form a semi-IPN structure. The resulting thermoset elastomers retained essential elastomeric characteristics while exhibiting improved thermal and mechanical performance. Successful formation of the semi-IPN structure was confirmed through comprehensive characterization, and the corresponding physical and thermal properties were systematically evaluated before and after thermal aging, along with gel fraction measurements. Combined results suggested that incorporating the liquid PI precursor was more effective than introducing solid monomers and solid/liquid mixture. Although the present findings do not yet demonstrate fully sufficient improvement, optimization of the precursor content within the NBR matrix via semi-IPN is expected to achieve the desired thermal performance without significantly compromising elastomeric properties.

## 1. Introduction

Acrylonitrile butadiene rubber (NBR) is a widely used synthetic elastomer in the automotive and petroleum industries due to its excellent oil resistance, relatively low cost, and good processability. However, the intrinsically low thermal stability of these materials significantly limits their application in high-performance systems that require resistance to temperatures above 120 °C [1,2,3]. Conventional strategies to overcome this limitation include sulfur-induced (i.e., S_8_ powder) vulcanization with reinforcing fillers (CB: carbon black, Zn^2+^, SiO_2_) to enhance overall physical properties via interconnected networks (ICNs) [4,5,6,7]. Separately, hydrogenation of the residual carbon–carbon double bonds (C=C) in the NBR backbone produces hydrogenated NBR (HNBR), which offers substantially improved thermal stability and service temperatures of up to 160 °C [8,9,10,11]. In the saturated NBR backbone, the absence of double bonds facilitates tighter chain packing and stronger intermolecular van der Waals interactions [9]. Similarly, chemical functionalization of nitrile groups in NBR to primary amide and carboxylic acid groups can facilitate the effective interactions between the functionalized NBR (XNBR) network and fillers to improve intermolecular interactions via ICNs [5,12]. Despite notable improvements, these modified NBR systems still fall short of fluoroelastomers (FKM with thermal stability up to 260 °C) [13,14,15], and HNBR/XNBR remain costly with processing limitations.

A more recent and cost-effective approach blends NBR with secondary high-performance polymers to access properties unattainable by NBR alone [16,17,18]. Such blended rubber systems can improve properties beyond what is achievable with neat NBR such as barrier performance, solvent resistance, and processability, while potentially reducing material costs through the use of complementary components. Polyimide (PI) is a particularly attractive candidate due to its outstanding thermal stability (>260 °C), high mechanical strength (101–128 MPa), and chemical resistance. Most reported NBR/PI composites, however, rely on simple physical blending of pre-formed PI powder into NBR [17,19,20,21,22], which is inherently limited by poor miscibility and phase separation between the polar PI and nonpolar NBR phases, often degrading mechanical consistency and elasticity [23,24]. As a result, despite its promise, PI has seen limited success as a reinforcing component in NBR systems, and only a few studies have achieved meaningful property enhancement through this blending strategy.

An alternative to overcome the miscibility limitations of physical blending is to form the PI network in situ, simultaneously with NBR vulcanization, rather than introducing pre-formed PI. In this approach, polyamic acid (PAA: a soluble, processable PI precursor) is compounded directly with NBR and curing agents in a single mixing step. During subsequent thermal treatment, NBR vulcanization (sulfur crosslinking) and PAA-to-PI imidization proceed concurrently within the same matrix, allowing the developing PI chains to interpenetrate the forming NBR network as it crosslinks, rather than being mechanically dispersed into an already-formed one. This simultaneous network development is expected to promote intimate physical entanglement between the two phases, mitigating the phase-separation issues that limit conventional PI-powder blends and yielding a semi-interpenetrating polymer network (semi-IPN: a crosslinked NBR network physically intertwined with linear PI chains) [2,5,25,26,27,28].

In this work, NBR/PI semi-interpenetrating polymer network (semi-IPN) composites were fabricated by directly incorporating either a PAA solution (liquid PI precursor) or a mixture of BTDA and ODA powders (solid PI precursors; benzophenone-3,3′,4,4′-tetracarboxylic dianhydride and 4,4′-oxydianiline, respectively) into an NBR compound during Banbury mixing. The compounds were then processed into sheets using a two-roll mill and thermally cured in a single step, during which sulfur-induced vulcanization of NBR and the condensation/imidization reactions to form PI occurred simultaneously. For comparison, conventional semi-IPNs were prepared by immersing crosslinked NBR in a PAA precursor solution, followed by thermal imidization. The PI loading efficiency and physicochemical properties of the resulting semi-IPNs were systematically compared. Additional post-curing further promoted PAA imidization regardless of whether the precursor was introduced as a liquid or solid. This one-pot simultaneous curing strategy was explored as a simple and scalable route for producing homogeneous NBR/PI semi-IPNs with improved thermal and mechanical properties while preserving the elastomeric characteristics of the NBR matrix.

## 2. Materials and Methods

### 2.1. Materials

The materials used in this study included the following NBR-related compounds: [base NBR mixture (KNV00726 with Mooney viscosity of 70–90 MU + KNB40H with Mooney viscosity of 80 MU, Kumho Petrochemical, Seoul, Republic of Korea), ZnO/stearic acid (Duksan Pure Chemical Co., Gyeonggi-do, Republic of Korea), N-isopropyl-N′-phenyl-p-phenylenediamine (IPPD)/poly(1,2-dihydro-2,2,4-trimethyl-quinoline) (RD, LANXESS Korea, Seoul, Republic of Korea), Sunoco 682 (DAEWOON, Gyeongsangnam-do, Republic of Korea), Mistron Vapor R (Choyang Chemical, Seoul, Republic of Korea), dibutoxyethoxyethyl adipate (TP-95, plasticizer, Seojin Chems Co., Gyeonggi-do, Republic of Korea), furnace black (carbon black N550, OCI company, Seoul, Republic of Korea), N-cyclohexyl-2-benzothiazolesulfenamide (CZ, LANXESS Korea), tetramethylthiuram disulfide (TT, LANXESS Korea), and S_8_ (Seyang Polymer, Incheon, Republic of Korea)]. In addition, 3,3′,4,4′-benzophenone tetracarboxylic dianhydride (BTDA, Aldrich, St. Louis, MO, USA) and 4,4′-oxydianiline (ODA, Aldrich, USA), and dimethylformamide (DMF, Duksan Pure Chemical Co., Republic of Korea) were purchased from the indicated vendors.

### 2.2. Preparation of Polyamic Acid (PAA)

**Figure 1:**
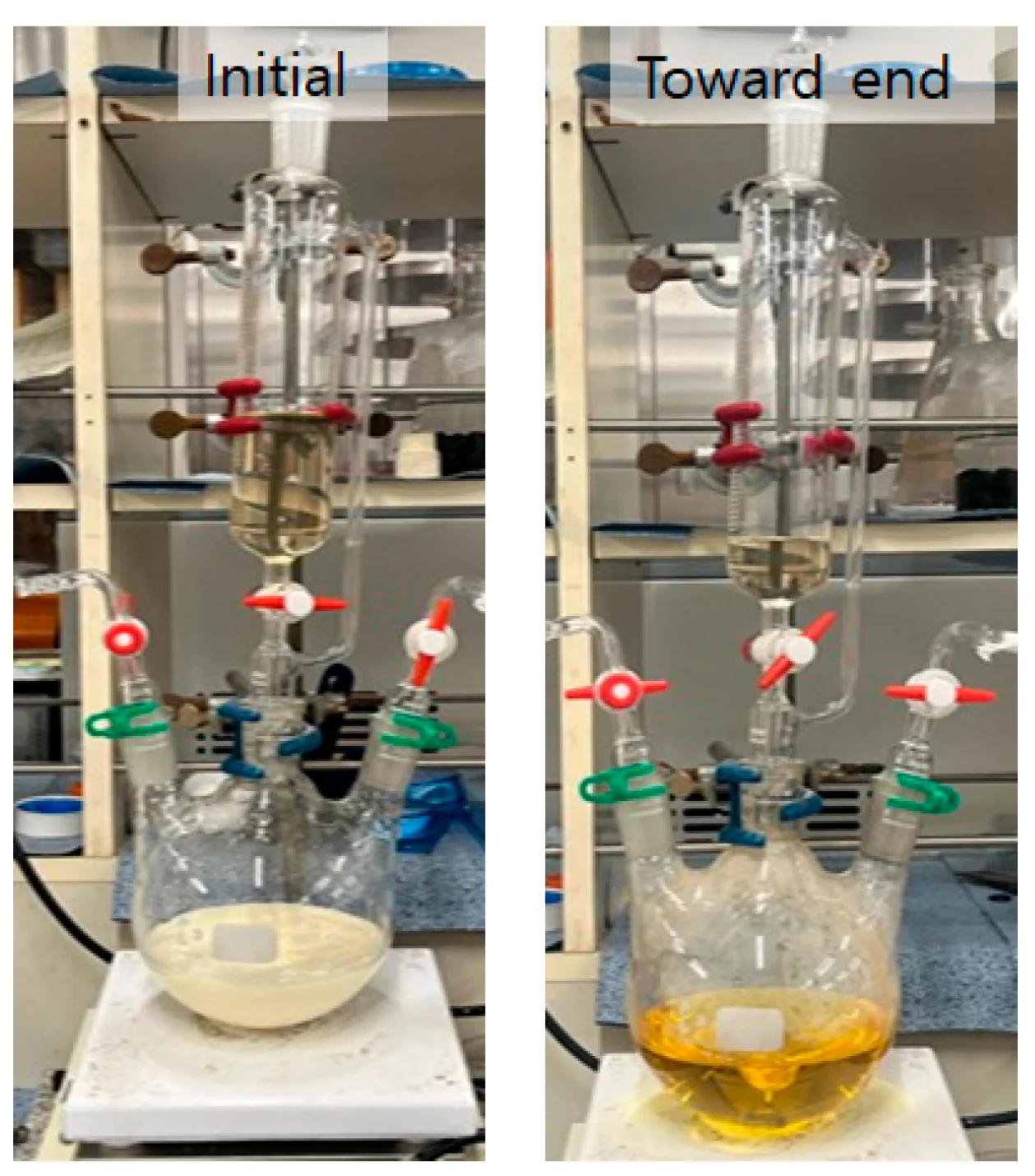
Digital photos illustrating the preparation process of the PI precursor (i.e., PAA) from BTDA and ODA monomers in DMF.

The liquid polyamic acid (PAA, a precursor to PI) was prepared by slowly mixing separately prepared BTDA and ODA in DMF under an inert atmosphere (Figure 1). Specifically, BTDA (30.5 g, 0.095 mol) was completely dissolved in 100 mL (94.5 g) of DMF in a 500 mL three neck round-bottom flask while purging with a N_2_ gas. In a separate flask, ODA (19 g, 0.095 mol) was dissolved in 100 mL of DMF at room temperature and transferred to a dropping funnel. After the dropping funnel was mounted onto the round-bottom flask, the entire system was purged with N_2_ gas for at least 30 min. Subsequently, the ODA solution was added dropwise over the course of hours where the initially turbid ivory BTDA solution changed to bright yellow, indicating the formation of polyamic acid (PAA). The mixture was stirred overnight to complete the condensation reaction, yielding a PI precursor solution with a concentration of ~25 wt%, based on the total monomer mass relative to the solvent mass. The viscosity of the as-prepared PAA solution (25 wt%) was measured using a rotational viscometer, yielding a viscosity of approximately 874 cP. The PAA solution was subsequently diluted to 0.05 wt in DMF, and flow times of the polymer solutions and pure solvent were recorded using a glass capillary Ostwald viscometer. The measured values were used to estimate the viscosity-average molecular weight (M*v*) of PAA using the Mark–Houwink equation, [η] = K$M_{v}^{a}$ [29,30,31]. Based on this approach, the M*v* of the PAA was estimated to be around 143,000 g/mol (detailed calculations for the viscosity parameters and M*v* estimation are provided in Figure S1 of the Supplementary Information). It is noted that as BTDA is somewhat susceptible to hydrolysis at room temperature, both the dissolution step and the subsequent condensation reaction were carried out under a N_2_ atmosphere throughout the entire process.

### 2.3. Formulation of NBR-PI Compounds

**Figure 2:**
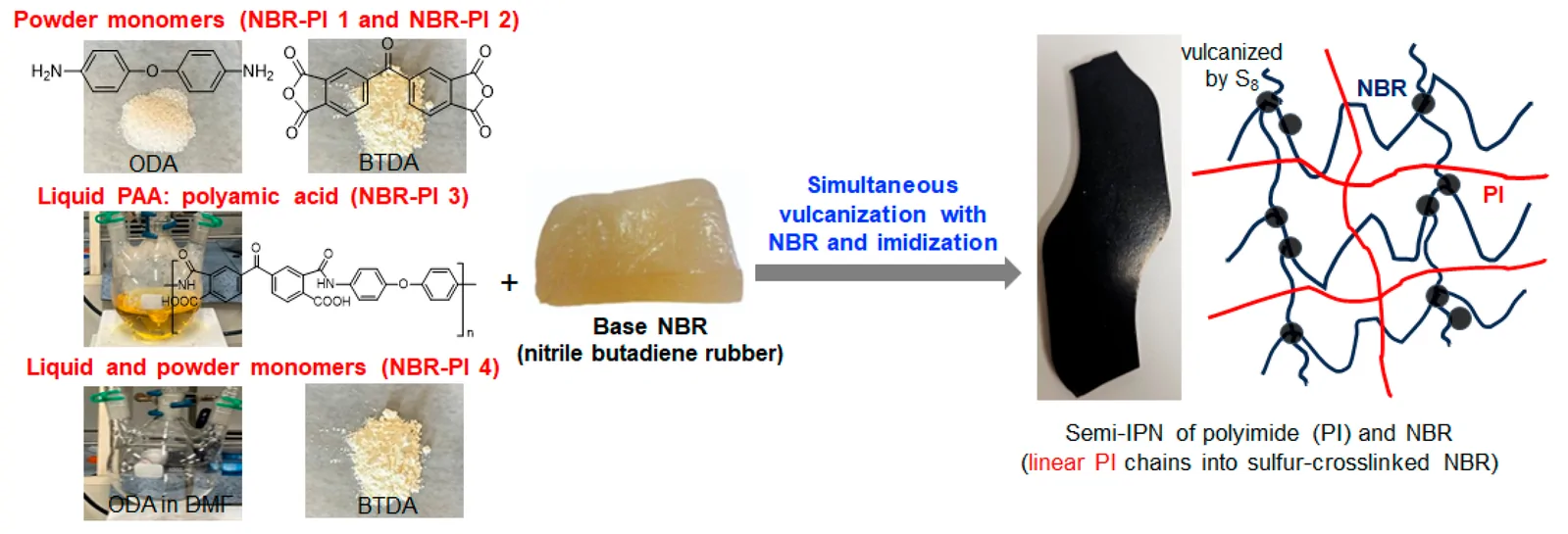
Preparation routes for various polyimide/NBR semi-IPN consisting of linear PI and crosslinked NBR.

The base NBR rubber used in this study consisted of KNV00726 (~33% medium-high acrylonitrile content) and KNB40H (~40% high acrylonitrile content) along with S_8_ powder as the vulcanizing/crosslinking agent. The following additional components were mixed with NBR and PI precursors: ZnO (activator), stearic acid (co-activator), IPPD/RD (antioxidants), Sunoco 682 (softening agent), Mistron Vapor R (high purity, platy talc to improve processability and performance), N 550 (carbon black reinforcer), TP-95 (plasticizer), CZ (primary accelerator), and TT (secondary accelerator). The detailed compounding formulation of the base rubber and additives are summarized in Table 1 and the representative process is shown in Figure 2.

Compounding, Rolling, and Vulcanization: Effective mixing of the rubber compounds was performed using a 360-mm Banbury Mixer (Hyunjin Machinery, Gyeonggi-do, Republic of Korea) at 50–80 °C for 5 min. Mastication was carried out in the same mixer at a fill factor of approximately 70% for 20 min to ensure uniform mixing. The compounded rubber was then sheeted on a 360 mm two-roll mill under the following conditions: roll ratio 1:1.25, roll speed 18 rpm, and roll gap (4 mm for initial and 2 mm for final). The resulting rubber sheets were conditioned at room temperature with a relative humidity below 65% for at least 24 h prior to vulcanization. Around 1.2 kg of master batch was prepared for each of the rubber formulations. Each batch yielded many tensile specimens and a minimum of eighteen compression specimens (Ø29 × 12.7 mm). These rubber specimens were subjected to a two-stage vulcanization process: a primary cure at 160 °C for 20 min, followed by a secondary cure at 180 °C for 1 h to achieve optimal crosslinking.

### 2.4. Characterization

The degree of crosslinking for the rubber composites were examined by a Mooney system equipped with a type of rotational viscometer (Vulchem IND Co., Gyeonggi-do, Republic of Korea). Based on ASTM D1646, all specimens were tested under identical conditions at 2 rpm at 121 °C (250 °F). Similarly, the torque value was examined by a moving die rheometer (RLR-3; rotorless rheometer, Toyoseiki, Japan) at a temperature of 160 °C and under an oscillation angle of ±1° according to ASTM D5289. After collecting the cure curves, several parameters including minimum torque (ML), maximum torque (MH), scorch time (ts_2_), and optimum cure time (tc_90_) were determined. The viscosity of the as-prepared liquid-phase polyamic acid (PAA) solution in DMF was determined using an advanced rotational viscometer (DVNext Rheometer, AMETEC Brookfield, Middleborough, MA, USA). For dilute PAA solutions, viscosity measurements were performed using a glass capillary Ostwald viscometer (1 mm Pin’s viscometer, Tianlian Group Technology Co., Beijing, China) to determine the specific/relative/intrinsic viscosity and estimate M*v* of the polymer precursor.

The physical property associated with hardness (Shore A) and overall mechanical properties (e.g., tensile strength, 100% modulus, and elongation at break) of the cured rubber were also examined by a Macro IRHD test system equipped with a Durometer A insert (Hilderbrand, Wendlingen, Germany) and a universal testing machine (UTM, KSU-05M-C, Kyoungsung Testing Machine Co., Gyeonggi-do, Republic of Korea), respectively. The hardness test of the rubber specimens was based on the Shore A hardness scale by measuring resistance to indentation at least five times according to ASTM D2240. The mechanical properties were evaluated according to KSM6518 (Methods for Physical Testing of Vulcanized Rubber) at 25 °C with a crosshead speed of 500 mm/min. For each rubber sample, the average values were obtained from a minimum of three rubber specimens. Dumbbell-type specimens were produced using an SD type lever-controlled sample cutter (SDL-200, Dumbbell Co., LTD., Saitama-Ken, Japan) according to ASTM D412 for these measurements.

For the aging test, the overall mechanical properties were also remeasured using the above UTM and compared with the original properties to evaluate heat resistance. Dumbbell-type rubber specimens were placed in an aging tester for 72 h, where heat waves were applied to keep the temperature constant at 150 °C or 200 °C. The Shore A hardness and mechanical properties were then measured using the same equipment and method described above (*vide supra*). The change was then calculated in comparison with the measurements before and after the aging test.

The oil resistance was evaluated using an oil resistance tester (TIG228267, Labtech Instrument Co., Guangdong, China) by immersing the rubber specimens in Fuel D (40% toluene and 60% isooctane) at 40 °C for 70 h. The mass change before and after immersion was recorded. Additionally, Shore A hardness, tensile strength, 100% modulus, and elongation were also measured and compared with the original properties to evaluate oil resistance.

All these analyses were based on the MS263-20 International Automotive material test standard [32], which can meet the Hyundai Motor Group specification for rubber materials used in heat-resistant, high-pressure hoses for vehicle heating and air intake systems [32,33].

Thermal properties of the rubber samples were evaluated by thermogravimetric analysis (TGA 55, TA Instruments, New Castle, DE, USA) and (DSC 25, TA Instruments, USA). The samples for TGA were around 5–10 mg in alumina pan under nitrogen conditions at a heating rate of 20 K/min from 25 to 700 °C. The samples for DSC were measured in an aluminum pan at a ramping temperature of 10 K/min from −40 °C to 300 °C. Surface morphology and elemental composition were examined by SEM–EDS using a JSM-IT510 InTouchScope™ (JEOL Ltd., Tokyo, Japan) at various magnifications. A small piece of rubber sample was mounted on carbon tape prior to imaging. Raman spectroscopy was utilized with a ProRaman-L (TSI, Shoreview, MN, USA), equipped with a 785 nm laser and a CCD detector (−50 °C). An objective (NA = 0.5) focused the 785 nm laser onto the rubber samples, and the laser power was adjusted to 10–12 mW. A Fourier-transform infrared spectrophotometer (FT-IR) equipped with an attenuated total reflection (ATR) sampling device (Spectrum 100 FT-IR Spectrometer, PerkinElmer, Shelton, CT, USA) was utilized to examine the presence of several functional groups across the rubber samples in the scan range of 600–4000 cm^−1^.

Lastly, a representative piece of each rubber sample was accurately weighed (initial dry mass, *W*_0_) and completely immersed in excess DMF at room temperature for 24 h to extract the soluble fraction. Following solvent extraction, the rubber samples were removed from DMF, gently wiped with paper towels to remove excess solvent, and dried to a constant weight. The final dry mass (*W_f_*) was then recorded, and the gel fraction was then calculated using the following equation to assess the overall crosslinking density: Gel fraction (%) = $W_{f}/W_{0}\times 100$ [34,35]. The same procedure was repeated once more using toluene as the extraction solvent for the bare NBR sample.

## 3. Results

### 3.1. Preparation Overview of Semi-Interpenetrating Polymer Network by Introducing Linear PI During the Vulcanization of NBR

**Scheme 1:**
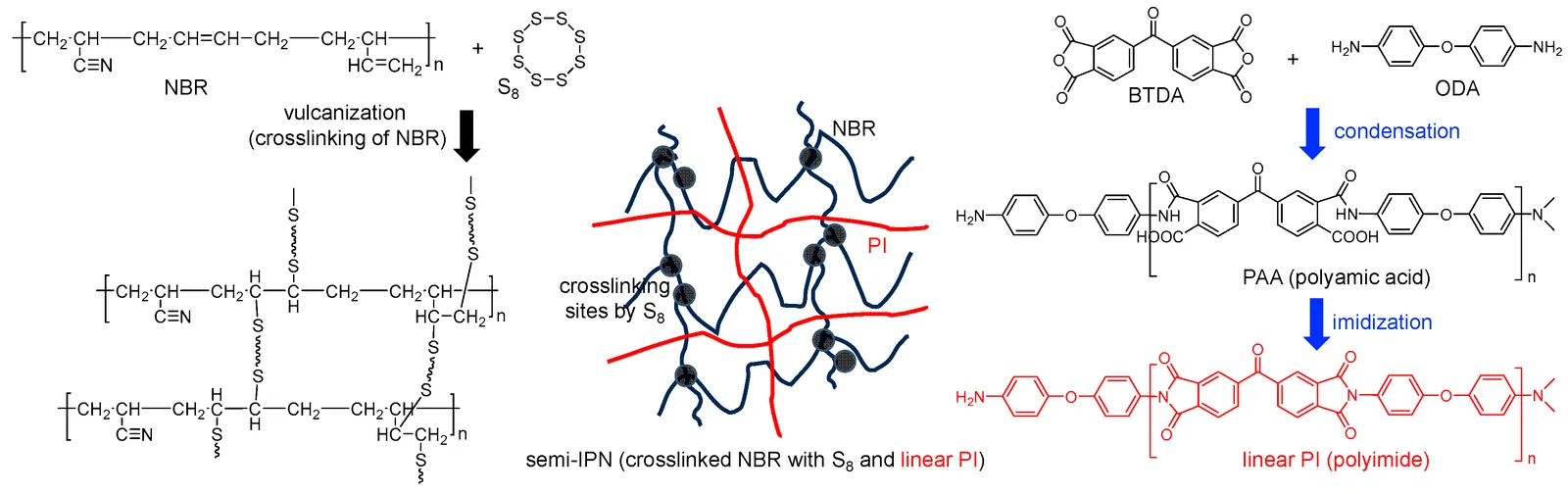
Structural illustration of a semi-IPN through NBR vulcanization with S_8_ and concurrent condensation polymerization of PI monomers (note: other fillers were excluded to clearly demonstrate the formation of the linear PI within the crosslinked NBR matrix).

The NBR-based rubber compounding was mainly completed via a two-stage process, which involves high-shear internal mixing of raw NBR with various additives including PI monomers or precursors in Banbury at 80 °C for around 15 min. Subsequently, the resulting compound from the Banbury was transferred onto two horizontal, counter-rotating metal rolls, where it was repeatedly passed through a narrow adjustable gap to produce a homogeneous sheet (i.e., promoting highly uniform mixing). The base NBR formulation meets the Hyundai Motor Group specification for heat-resistant, high-pressure hoses used in vehicle heating and air intake systems. The preparation of a semi-interpenetrating polymer network was initially achieved by introducing solid monomers and solid/liquid mixture, or PI precursors (i.e., PAA—polyamic acid) during the compounding process of NBR base mixture (Scheme 1). During vulcanization, the NBR matrix underwent crosslinking with S_8_, while simultaneous condensation reactions took place to form PAA, followed by further imidization to yield a linear PI. Thus, the integration of PI units possessing exceptional thermal stability and mechanical strength across the NBR matrix could significantly impact the overall physicochemical properties of the composite rubber materials.

### 3.2. Characterization of Unvulcanized Rubber Compounds

The initial NBR-based rubber compound sheets were evaluated by a Moving Die Rheometer (MDR) and a Mooney viscometer. MDR measurement is a key analytical step for assessing the cure characteristics of vulcanizable rubber samples by examining curing rate, scorch time, cure density, and processability according to ASTM D5289 at 160 °C. A Mooney viscometer equipped with a rotating shear disk is a standard testing approach for determining the viscosity (Mooney viscosity) and scorch behavior (e.g., T_5_) of unvulcanized rubber compounds. The rubber specimens were prepared according to the procedures described in ASTM D3182. Based on these two tests, the results were summarized in Table 2. The overall characteristics of NBR–PI 1 were comparable to those of the commercial NBR, whereas those of NBR–PI 3 were slightly higher. On the other hand, NBR–PI 2 exhibited the highest apparent crosslinking density, but the NBR–PI 4 composite samples showed unusual signs of incomplete vulcanization, which was evidenced by the relatively slower cure times (initial ts_2_ and optimum tc_90_) and the lower degree of crosslinking density (T_max_ − T_min_). For example, a larger torque difference (ΔT) generally indicates a higher apparent crosslinking density (e.g., as an indirect indicator of the extent of vulcanization/crosslink development). Additionally, relatively higher Mooney viscosity and notably longer T_5_ values further suggested somewhat incomplete curing in the NBR-PI 1 and NBR-PI 2 samples. In contrast, NBR-PI 3 and NBR-PI 4 displayed similar curing characteristics, with slightly improved processability compared to the NBR control sample.

### 3.3. Morphological Features of Vulcanized Rubber Compounds

**Figure 3:**
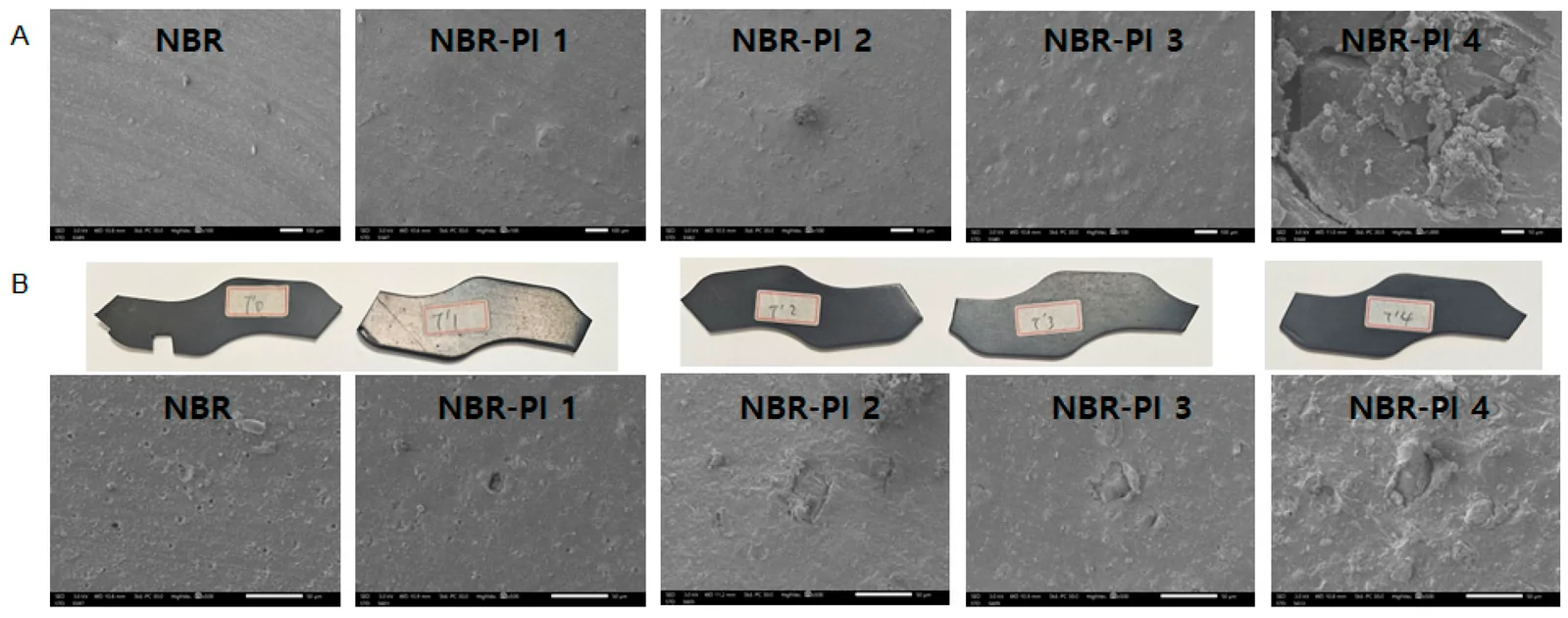
SEM images of NBR and NBR-PI semi-IPNs after the initial vulcanization at 160 °C (**A**) and final vulcanization (**B**) of NBR-based composite rubbers at 180 °C and corresponding digital photos (T′0 for the NBR control and T′1-T′4 for NBR-PI 1 to NBR-PI 4). Note: scale bar = 10 μm.

After examining the compounds, these rubber sheets were vulcanized at elevated temperatures (160 °C for 20 min and 180 °C for 1 h). This step simultaneously induced the condensation polymerization of PI monomers and imidization of PAA to form linear PI across the crosslinked (/vulcanized) NBR. Digital photos and cross-section images of these composite rubber samples were examined by scanning electron microscopy to understand how the semi-IPN structural evolution was influenced by the way the PI monomers introduced (Figure 3). The rubber samples vulcanized at 160 °C exhibited somewhat smoother and more even morphologies, whereas those treated at 180 °C for 1 h started showing the formation of noticeable voids and somewhat uneven structural patterns. These hole-shape detects could be due to the release of entrapped gaseous species or water molecules generated during the condensation reactions in the vulcanization process. Moreover, the NBR-PI 4 sample, prepared with a mixture of BTDA powder and liquid ODA in DMF, resulted in the least uniform morphological feature, which might be attributed to relatively poor miscibility within the base NBR matrix. This observation may be related to the coexistence of solid and liquid phase components within a rigid NBR matrix, hindering effective mixing. In contrast, it is noted that NBR-PI-1 and NBR-PI-2 were prepared using BTDA and ODA in powder form, while NBR-PI-3 employed a liquid PAA (i.e., PI precursor).

**Table 2:** Characterization of unvulcanized rubber compounds using a rheometer and Mooney viscometer.

	Test Name	Test Spec	NBR (Control)	NBR-PI 1	NBR-PI 2	NBR-PI 3	NBR-PI 4
Unvulcanized compounds	Rheometer (160 °C × 12 min)	T_max_ (N⋅m)	0.82	1.02	1.66	0.85	0.35
		T_min_ (N⋅m)	0.18	0.42	0.50	0.14	0.15
		tc_90_ (minutes)	3.98	10.68	10.39	3.83	10.67
		ts_2_ (minutes)	2.54	5.68	2.45	2.03	11.69
	Mooney Viscosity (121 °C)	MV	41.00	74.40	91.70	36.90	34.40
		T_5_ (minutes)	16.30	87.55	68.59	11.15	50.47

### 3.4. Mechanical Properties of Vulcanized Rubber Compounds

Subsequently, the mechanical properties of the resulting composite rubbers were then thoroughly examined by universal testing machine (UTM) and Macro IRHD tester (Table 3). The three semi-IPN composite samples prepared with PI powder monomers (i.e., NBR-PI 1, NBR-PI 2, and NBR-PI 4) generally did not meet the recommended minimum specification. Interestingly, these rubbers also exhibited somewhat larger variations in mechanical properties, possibly caused by relatively uneven or limited distribution of the powder monomers across the base NBR matrix. However, the semi-IPN prepared from the liquid PAA (i.e., NBR–PI 3) achieved improved dispersity within the NBR network, resulting in more consistent properties and overall performance that met the required criteria, albeit with slightly reduced hardness. These findings suggest that successful integration of linear PI into crosslinked NBR to form semi-IPNs is strongly dependent on the method of introducing PI monomers or precursors, which in turn significantly influences the overall mechanical properties.

### 3.5. Characterization of PI Formation During the Vulcanization of NBR

**Figure 4:**
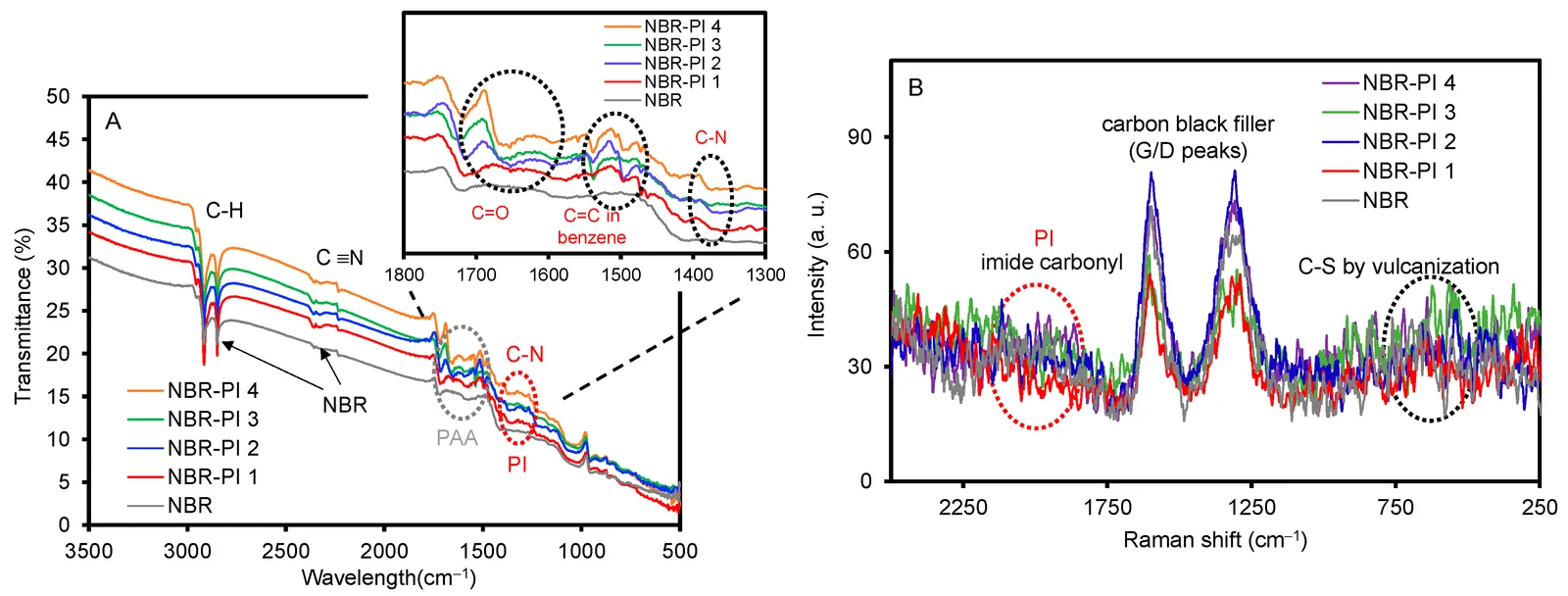
FT-IR (**A**) and Raman (**B**) spectroscopy of vulcanized NBR and NBR-PI semi-IPNs.

To confirm the formation of PI within the NBR network, Raman and IR spectrophotometers were employed to analyze characteristic vibrational peaks and compare pristine NBR with PI-containing rubber composites (Figure 4). Strong characteristic peaks of NBR functional groups appeared at 2900 cm^−1^ for asymmetric CH_2_ and 2240 cm^−1^ for CN stretching, respectively [36]. Upon the incorporation of linear PI, the resulting NBR-PI semi-IPNs displayed an additional detectable peak at ~1380 cm^−1^ for characteristic imide C-N stretching, along with broad bands in the 1600–1700 cm^−1^ region, likely arising from C=O and N-H groups associated with residual PAA. Similarly, the Raman spectrum of vulcanized NBR mainly showed a crosslinked network by sulfur linkage at ~600 cm^−1^ and two strong peaks at 1250–1750 cm^−1^ for typical carbon black fillers with graphitic (G) and defect (D) domains [37]. The G and D peak area may overlap with contributions from C=C stretching (unsaturated) in NBR and benzene ring of PAA/PI (~1540 cm^−1^) and C-N stretching in PI (1385 cm^−1^). Notably, all four NBR-PI semi-IPNs exhibited a detectable peak at 1800 cm^−1^, which is probably attributed to imide C=O stretching and appears at a slightly higher wavenumber than the ~1700 cm^−1^ peak associated with the C=O stretching of PAA (-COOH) groups [38,39]. To clearly distinguish the characteristic spectral features before and after the imidization of PAA to PI within the NBR matrix, a similar reaction was also carried out in the absence of NBR (Figure S2). The FT-IR spectra clearly confirmed the successful conversion of PAA to PI, as evidenced by changes in the broad OH over 3000 cm^−1^, the C=O and C-NH bands in the 1660–1710 cm^−1^ region, and the C-N peak at ~1380 cm^−1^. In contrast, the Raman spectra showed less pronounced spectral changes (e.g., growth of the imide ring, symmetric C=O stretching, and PI ring deformation) under the experimental conditions used. These observations are consistent with previous reports, which have shown similar FT-IR and Raman spectral evolution during the conversion of PAA to PI, even at imidization temperatures below 180 °C [40,41,42]. Moreover, the digital photos of the resulting powders revealed a color change from light yellow to dark yellow, providing additional visual evidence for the successful formation of PI. Overall, these vibrational spectroscopy analyses provided supporting evidence for the successful formation of PI within the vulcanized NBR network.

**Figure 5:**
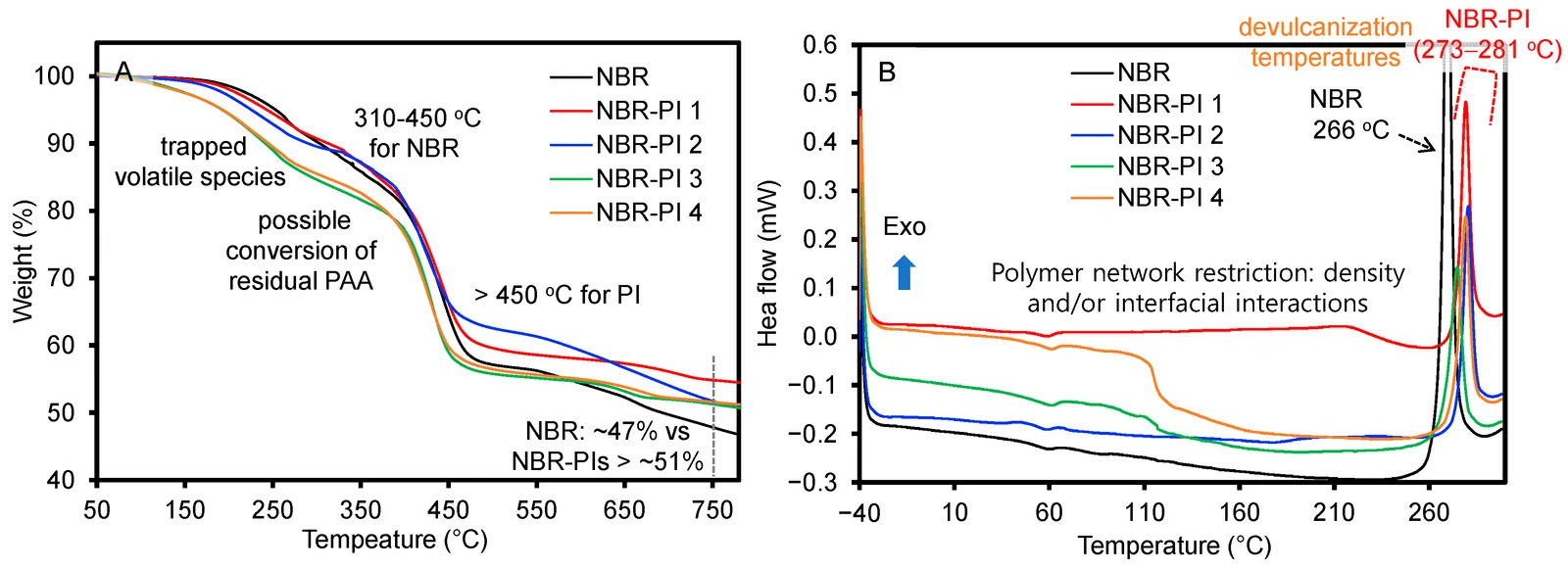
TGA (**A**) and DSC (**B**) thermograms of vulcanized NBR and NBR-PI semi-IPNs.

To further investigate the incorporation of PI, thermogravimetric analysis (TGA) and differential scanning calorimetry (DSC) were performed (Figure 5). The overall degradation patterns by the thermograms were comparable across the entire temperature range. The initial weight loss below 200 °C could be related to the removal of entrapped volatile species, while the major degradation at slightly over 310 °C corresponded mainly to the decomposition of the NBR matrix. Continuous weight loss above 450 °C was possibly associated with further degradation of the NBR phase. In comparison, all PI-containing NBR samples exhibited a slightly slower and more gradual weight loss behavior. Notably, the final weight loss (%) of the vulcanized NBR was detectably higher than that of the PI-containing NBR samples, with the latter retaining more than 50 wt% residue. Among the NBR-PI semi-IPNs, distinct differences in thermal behavior were observed between samples prepared using solid PI monomers and those prepared using liquid precursors. Specifically, the preparation of NBR-PI 1 and NBR-PI 2 involved the imidization reaction using powder monomers (i.e., solvent-free environment) via mechanochemistry, which could promote high conversion by a direct reaction. On the other hand, NBR-PI 3 and NBR-PI 4 samples were prepared with at least one monomer or PAA precursor suspended in a solvent medium (i.e., imidization in solvent), resulting in a more heterogeneous reaction environment with other stabilizing reactants, which could inhibit the overall conversion yields. While the solvent may facilitate molecular mobility and collision frequency, it can also stabilize reactants and require higher thermal input to achieve complete imidization. This speculation partially explained the TGA weight loss pattern where the NBR-PI 3 and NBR-PI 4 samples lost more volatile species below 200 °C and possibly ongoing imidization over 200 °C (i.e., conversion of PAA to PI) and the concomitant release of water molecules [43,44,45]. In this aspect, one can speculate that NBR-PI-1 and NBR-PI-2, prepared via mechanochemistry, did not exhibit noticeable additional weight loss in these temperature regions, consistent with a more direct and complete imidization process.

Separately, the entrapped DMF in the rubber samples was removed by heating the samples at 100 °C for 30 min prior to TGA analysis to minimize the influence of residual solvent and enable a more accurate examination of the resulting thermograms (Figure S3). The overall degradation pattern remained very similar to the rubber samples. However, compared with the NBR, the semi-IPNs resulted in a detectable increase in both the decomposition temperature at 5 wt% and the residual rubber content measured at 700 °C with respect to the NBR. Specifically, the initial decomposition temperature increased from 364 °C to 368 °C, while the residual rubber content increased from 42 wt% to 46 wt%. These results suggest that the incorporation of PI slightly enhanced the thermal stability of the NBR-based material and promoted the formation or retention of thermally stable components during thermal degradation. A similar trend was previously reported for blended NBR/PI composite rubbers, further supporting the observed effect of PI incorporation [17]. This finding suggested that the detectable differences in the TGA thermograms could provide meaningful insight into the effects of DMF presence or absence on the thermal stability of the rubber samples and contribute to their interpretation.

In addition, DSC thermograms did not show discernible glass transition temperatures for all samples, despite the use of a low heating rate starting from −40 °C. However, a distinct difference in heat flow was observed corresponding to the devulcanization temperature of NBR. It has been reported that neat NBR typically exhibits devulcanization at near 200 °C [46,47,48]. Our control sample (i.e., vulcanized NBR) was formulated based on a commercial grade used for Hyundai automotive hose specifications where the incorporation of additional fillers, including carbon black and ZnO, can form interconnected networks (ICN) between NBR matrix and these fillers, resulting in a significant increase in the devulcanization temperature. The formation of such ICNs during rubber compounding greatly contributes to enhanced composite performance by establishing continuous, physically linked structures in which filler–filler interactions effectively reinforce the rubber matrix. Thus, this NBR with these additional fillers displayed a much elevated devulcanization temperature of around 266 °C. Furthermore, all PI-containing NBR (semi-IPNs) exhibited further increased devulcanization temperatures in the range of 273–281 °C, indicating that the incorporation of linear PI enhanced the thermal stability of the vulcanized NBR. This improvement can be attributed to increased interfacial interactions between PI and the NBR network via physical engagement and intermolecular forces [49,50].

To provide direct evidence for the semi-IPN structure, gel fraction measurements were performed by extracting vulcanized and imidized rubber samples in an organic solvent for 24 h and calculating the ratio of final to initial dry mass (Figure S4). Digital photos show the visibly reversible swelling and deswelling texture of all specimens immediately before and after removal from solvent further confirming that each rubber network underwent substantial solvent uptake prior to extraction, consistent with a crosslinked rather than fully soluble structure across all six samples. Neat NBR extracted in toluene and DMF gave closely matching gel fractions (87.4% and 87.9%, respectively). Thus, the choice of DMF as the extraction solvent for the NBR-PI composite samples does not influence the gel fraction results (e.g., no solvent effects from the main NBR rubber). All four NBR-PI samples showed substantially higher gel fractions than neat NBR (91.5–95.2% vs. ~87–88%). Although one can speculate that the linear PI component could be freely released from the NBR matrix, this observation evidently indicates that a significant fraction of the PI component is entrapped within the crosslinked NBR network, directly confirming semi-IPN formation (i.e., physical entanglement of PI and NBR). Notably, the degree of crosslinking correlates with the rheometry data we discussed above. Specifically, NBR-PI 2 displayed the highest gel fraction (95.2%), consistent with its markedly high ΔT (T_max_ − T_min_) from the above cure rheometer data, which indicated the highest crosslink density among all formulations. Similarly, NBR-PI 3 showed the smallest gel fraction increase relative to control (90.7%), paralleling its cure behavior, which most closely resembled that of neat NBR across T_max_, T_min_, tc_90_, and ts_2_, further supporting the interpretation that this sample contains a relatively low PI content.

### 3.6. Thermal Aging and Oil Resistance Properties of Vulcanized NBR and NBR-PI Semi-IPNs

After confirming the successful integration of linear PI into NBR in the form of a semi-IPN, thermal aging tests were carried out at 150 °C for 72 h. Although thermal aging of NBR-derived hoses is often performed according to ASTM D573 (100–120 °C in an air circulating oven for several days), nonstandard higher temperature conditions were employed here to better assess the influence of PI in semi-IPN structures. As expected, the vulcanized NBR control sample exhibited cracking and severe defects, which prevented tensile strength and elongation measurements by UTM. In contrast, all PI-containing NBR samples retained their original shape, enabling mechanical and hardness testing. However, except for NBR-PI-3, the semi-IPN samples showed only marginal changes in tensile strength and a notable reduction in elongation, along with a slight increase in hardness, indicating limited suitability for practical applications under these harsh conditions. It appears that the presence of linear PI within the vulcanized NBR network partially helped preserve structural integrity of rubber even under more severe thermal treatment conditions (i.e., 200 °C for 72 h). Despite this stabilizing effect, pronounced thermo-oxidative degradation, significant hardening, and reduced elongation were still observed, suggesting that further optimization is required.

Similarly, oil resistance tests were also evaluated using Fuel D (a standard test fuel comprising 60% isooctane and 40% toluene) to assess the suitability for fuel hoses and sealing applications (Table 4). Hardness, mechanical properties, and volume changes were systematically monitored and compared with the vulcanized NBR control. Since oil resistance is closely related to acrylonitrile content, only slight reductions in resistance were observed, but all values remained within acceptable specification ranges. Notably, the NBR-PI-3 sample exhibited comparable or slightly improved performance relative to the vulcanized NBR control.

In addition, conventional semi-IPNs were prepared using a modified soaking method with a PAA precursor solution (Figure S5). Specifically, vulcanized NBR rubber specimens were fully immersed in the PAA solution, followed by in situ thermal imidization to generate the linear PI across the crosslinked NBR network. The resulting NBR-PI semi-IPN exhibited detectably meaningful improvements in overall mechanical properties. However, this soaking approach readily allowed only a limited uptake of PAA, corresponding to approximately 5–7 wt% within the NBR matrix under our preparation conditions. It should also be noted that the soaking approach based on the direct use of the monomers BTDA and ODA is highly impractical because BTDA is sensitive to moisture and readily undergoes hydrolysis upon exposure to air, particularly under humid conditions. Moreover, the soaking process is not well suited for industrial-scale production because of its low monomer loading efficiency and the requirement for multiple processing steps. In contrast, the approach developed in this study is not constrained by the incorporation efficiency of either the monomers or the PAA precursor. More importantly, the simultaneous crosslinking and polymerization processes enable the facile preparation of semi-IPN composites and offer a more practical route toward the scalable production of a wide range of rubber-based composite materials.

Although the present findings do not yet demonstrate fully satisfactory improvement, further optimization of the liquid PAA precursor content within the NBR matrix is expected to deliver the desired thermal performance without significantly compromising elastomeric properties. The development of such thermally stable rubber materials, while preserving their physical and chemical performance, could broaden their applicability across diverse industrial sectors and provide a promising alternative to high-cost fluoroelastomers (FKM) rubber materials, which are also associated with serious environmental and human health concerns.

## 4. Conclusions

This work demonstrates the feasibility of developing thermally stable NBR-based semi-interpenetrating polymer networks (semi-IPNs) through the incorporation of linear PI with enhanced heat resistance. Although the present semi-IPNs do not yet achieve the thermal stability of commercial fluoroelastomers (FKMs) or other high-performance heat-resistant rubbers (e.g., HNBR, XNBR), the PI/NBR semi-IPNs potentially exhibit a clear improvement over pristine NBR, as evidenced by the increased devulcanization temperature and gel fraction measurements, while maintaining acceptable elastomeric behavior and mechanical properties. Compared with conventional rubbers designed for high-temperature applications, the semi-IPN strategy offers several advantages, including a simple fabrication process, the use of commercially available monomers and readily synthesized precursors, the potential for lower material costs, and the elimination of fluorinated components. However, further optimization of the PAA precursor content, dispersion, and molecular weight, as well as the degree of PAA imidization and the resulting semi-IPN network structure, is still required to achieve more precise control over the materials’ properties and improve overall thermal stability without compromising the desirable physical and chemical characteristics of NBR. Thus, this study provides practical and scalable insights into the development of heat-resistant NBR materials and suggests that this IPN strategy may offer a promising alternative for a wide range of industrial applications requiring improved thermal stability, with the potential to reduce reliance on FKM-based rubber materials associated with environmental and human health concerns.

## Figures and Tables

**Table 1:** Formulation of rubber compounds to prepare semi-interpenetrating polymer networks.

Ingredients	Actual Quantity (g)					
	Parts per hundred rubber (phr)	NBR (Control)	NBR-PI 1	NBR-PI 2	NBR-PI 3	NBR-PI 4
Base NBR (KNV00726 + KNB40H)	100	600	545	500	500	505
ZnO/Stearic acid IPPD/RD	5/1 1/1	30/6 6/6	27.5/5.5 5.5/5.5	25/5 5/5	25/5 5/5	25/5 5/5
Sunoco 682 Mistron Vapor R	2 30	12 180	11 164	10 150	10 150	10 152
N 550	40	240	218	200	200	202
TP-95	15	90	82	75	75	76
CZ/TT	2/1.5	12/9	11/8	10/7.5	10/8	10/7.6
S_8_	0.5	3	2.7	2.5	2.5	2.7
BTDA/ODA powder PAA (~25 wt%) in DMF BTDA powder/ODA in DMF			67/42 (~20 phr)	123/77 (~40 phr)	50 (~10 phr)	67/42 (~20 phr)

**Table 3:** Mechanical properties of vulcanized NBR and NBR-PI semi-IPNs.

Test Name	Test Spec	SPEC MS263- 20AA1	NBR (Control)		NBR-PI 1		NBR-PI 2		NBR-PI 3		NBR-PI 4	
Physical properties after vulcanization (1st:160 °C × 20 min 2nd:180 °C × 1 h)			1st	2nd	1st	2nd	1st	2nd	1st	2nd	1st	2nd
	Hardness (Shore”A”)	73 ± 5	69	73	85	90	88	95	62	77	64	87
			±3	±3	±3	±4	±3	±4	±2	±3	±2	±3
	Tensile strength (MPa)	9.81 minimum	11.87	12.45	4.41	7.94	5.39	7.65	8.43	9.81	2.26	5.59
			±0.46	±0.48	±0.17	±0.31	±0.21	±0.30	±0.33	±0.38	±0.09	±0.22
	Elongation (%)	230 minimum	668	575	331	229	287	113	507	403	542	230
			±26	±22	±13	±9	±11	±4	±20	±16	±21	±9

**Table 4:** Aging and oil resistance tests of vulcanized NBR and NBR-PI semi-IPNs.

Test Name	Test Spec	SPEC MS263- 20AA1	NBR (Control)		NBR-PI 1		NBR-PI 2		NBR-PI 3		NBR-PI 4	
Aging test (150 °C × 72 h)	Hardness (Shore “A”) change	Non-standard test	+29	+26	+13	+8	+10	+4	+32	+18	+33	+10
	Tensile strength change (%)		Not applicable		+221	+81	+175	+107	+9	+8	+337	−90
	Elongation (%)				−48	−82	−87	−76	−89	−93	−96	−96
Oil resistance test (A) (40 °C × 70 h × Fuel D)	Hardness (Shore “A”)	−30~0	−34	−38	−47	−38	−37	−21	−22	−35	−34	−38
	Tensile strength change (%)	−45	−31	−37	−66	−52	−64	−48	−33	−40	−52	−56
	Elongation change (%)	−40	−23	−35	−37	−19	−49	−12	−28	−30	−54	−27
	Volume change (%)	+35	+36	+40	+37	+37	+32	+33	+31	+40	+33	+41

## Data Availability

The original contributions presented in this study are included in the article. Further inquiries can be directed to the corresponding authors.

## Supplementary Materials

The following supporting information can be downloaded at: https://www.mdpi.com/article/10.3390/polym18182267/s1, Figure S1. A photo of the capillary Ostwald viscometer setup and calculation of the viscosity-average molecular weight (M*v*) of the synthesized polyamic acid (PAA) using the Mark–Houwink relationship; Figure S2. Digital photos presenting PAA and PI powder preparation in the absence of NBR along with corresponding Raman (A) and FT-IR (B) spectroscopy (note: The small-scale imidization reaction without a reflux condenser often resulted in significant solvent loss and premature solidification of the reaction mixture); Figure S3. TGA thermograms of NBR and NBR–PI semi-IPNs after DMF solvent removal by heating at 100 °C for at least 30 min; Figure S4. Digital photographs of the rubber samples before and after gel fraction measurements, along with the corresponding gel fractions (%); Figure S5. Conventional preparation of NBR-PI semi-IPNs via a modified soaking method (A), along with cross-sectional images and mechanical properties of vulcanized NBR and NBR-PI semi-IPNs (B).
