# Supplementary material for: Impact of Polyimide Semi-Interpenetrating Polymer Networks on the Physicochemical Properties of Vulcanized Acrylonitrile–Butadiene Rubber
Source: Polymers (Basel). 2026 Sep 17;18(18):2267. doi: 10.3390/polym18182267 (PMC13611403; doi:10.3390/polym18182267)
Supplement: Supplementary file 1 [file polymers-18-02267-s001.zip › polymers-4513142-supplementary.pdf]

polymers

MDPI

Supplementary Material

Impact of Polyimide Semi-Interpenetrating Polymer Networks on the Physicochemical Properties of Vulcanized Acrylonitrile–Butadiene Rubber

Sunhyeong Kwon [1], Ji Min Kim [1], Geun-Su Park [2], Chul-Young Kim [2], Garrett M. Milligan [3], Jian Hou [4], Zhicheng Cai [5], Jun-Hyun Kim [3,*] and Hyun-Ho Park [1,*]

[1] Department of Chemistry, Keimyung University, Daegu 42601, Republic of Korea; gikme001@naver.com (S.K.); qudaktaosldk@naver.com (J.M.K.)
[2] R&D Centers, Pyung Hwa Industrial Co., Ltd., Daegu 42982, Republic of Korea; power79@ph.co.kr (G.-S.P.); cykim@ph.co.kr (C.-Y.K.)
[3] Department of Chemistry, Illinois State University, Normal, IL 61790, USA; gmillig@ilstu.edu
[4] School of Intelligent Manufacturing, Luoyang Institute of Science and Technology, Luoyang 471023, China; jhou@lit.edu.cn
[5] Department of Semiconductor System Engineering, Sejong University, Gwangjin-gu, Seoul 05006, Republic of Korea; cai1121@sejong.ac.kr
* Correspondence: jkim5@ilstu.edu (J.-H.K.); rubchem@kmu.ac.kr (H.-H.P.)

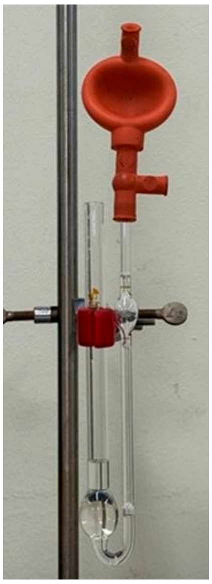

Flow time for DMF: 7.27 ± 0.06
Flow time for diluted PAA solution (0.05 wt%): 8.13 ± 0.06
$\eta_{relative} = 8.13/7.27 = 1.118$
$\eta_{specific} = 1.118 - 1 = 0.118$
$\eta_{reduced} = 0.118/[0.05] = 2.36$ dL/g

As an approximation, the reduced viscosity ($\eta_{reduced}$) was used as the reduced viscosity ($\eta_{intrinsic}$) as the PAA concentration (0.05 wt%) was close to near zero, where intrinsic viscosity is defined.

Given the Mark-Hounwink equation: $[\eta] = K M_v^{\alpha}$, where M*v* = viscosity-average molecular weight, K = 1.85 x 10^{-4} dL/g, and $\alpha = 0.8$ (these two parameters are adopted from a standard aromatic PAA system derived from pyromellitic dianhydride and 4,4'-oxydianiline).

[2.36 dL/g] = (1.85 x 10^{-4} dL/g) $M_v^{0.8}$ → M*v* ≈ 136,000 g/mol

Figure S1. A photo of the capillary Ostwald viscometer setup and calculation of the viscosity-average molecular weight (M*v*) of the synthesized polyamic acid (PAA) using the Mark–Houwink relationship.

A

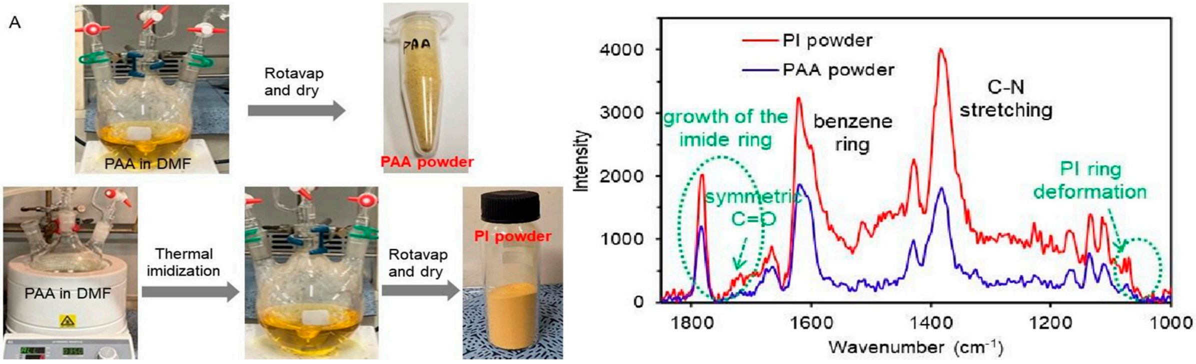

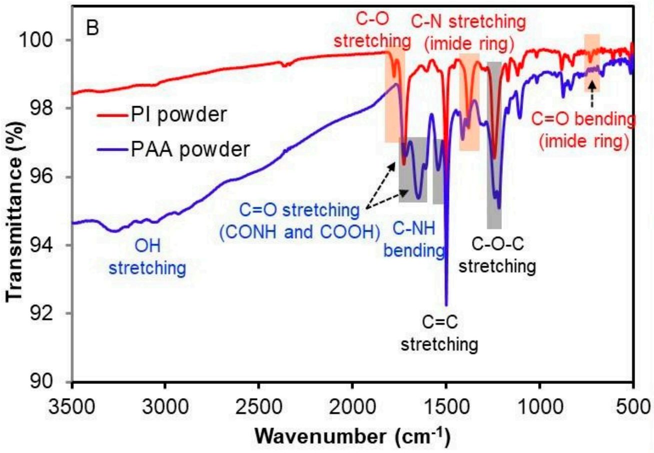

	cm^{-1}	Functional group
PI	~1780 (medium)	V_{as} C=O stretch
	~1720 (strong)	V_s C=O stretch
	~1380 (strong)	Vs C-N stretch in imide ring
	~726 (weak)	C=O bending in imide ring
PAA	> 3000	O-H (COOH)
	~1710 (strong)	C=O (COOH)
	~1660 (strong)	C=O (CONH)
	~1550 (medium)	C-NH bending
	~1500 (strong)	V_s C=C in benzene

Figure S2. Digital photos presenting PAA and PI powder preparation in the absence of NBR along with corresponding Raman (A) and FT-IR (B) spectroscopy (note: The small-scale imidization reaction without a reflux condenser often resulted in significant solvent loss and premature solidification of the reaction mixture).

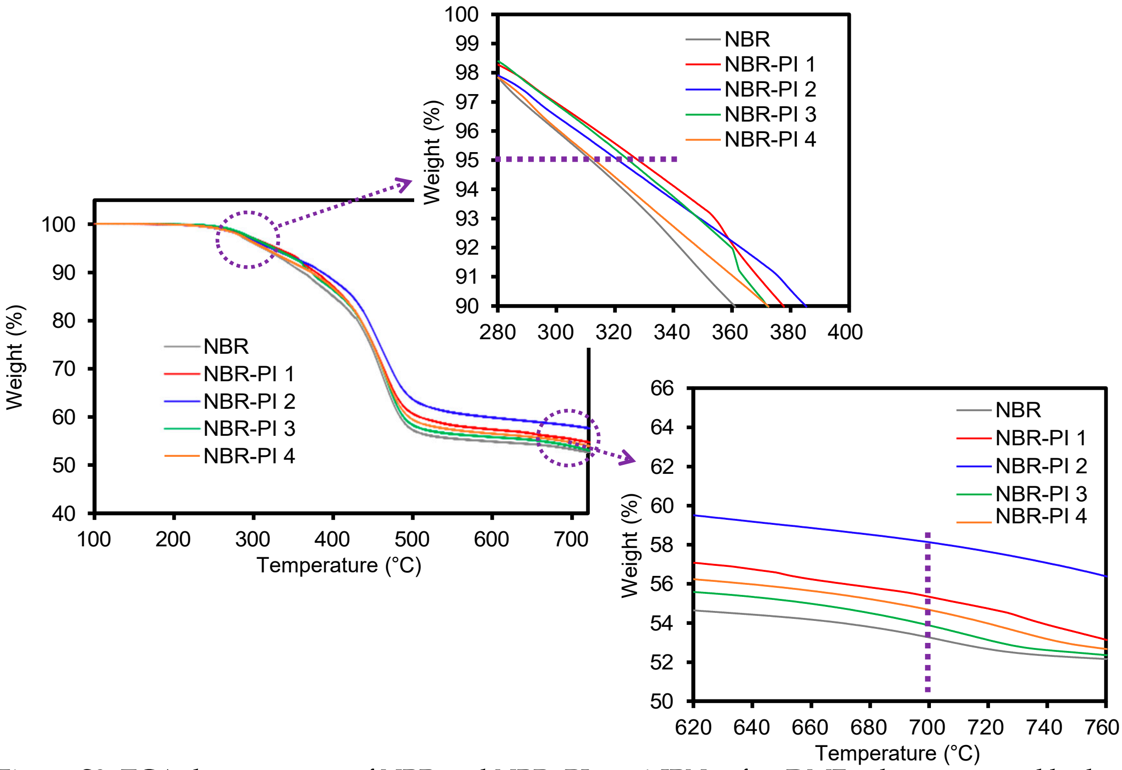

Figure S3. TGA thermograms of NBR and NBR–PI semi-IPNs after DMF solvent removal by heating at 100 °C for at least 30 min.

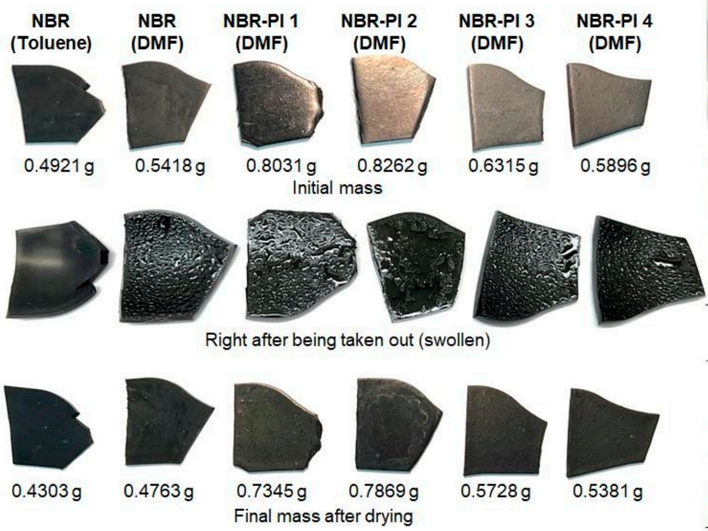

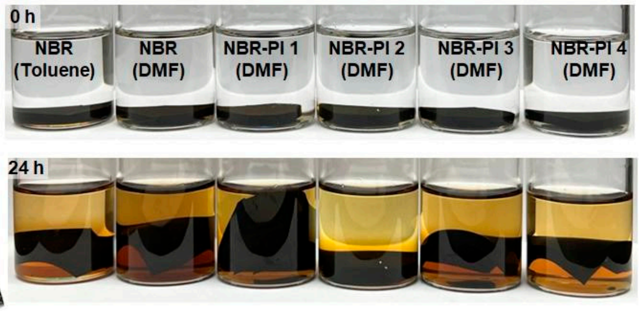

Sample	Initial Mass (g)	Mass after air drying (g)	Mass after oven drying (g)	Gel Fraction (%)
NBR (Toluene)	0.4921	0.4614	0.4303	87.4
NBR (DMF)	0.5418	0.6614	0.4763	87.9
NBR-PI 1 (DMF)	0.8031	0.8404	0.7345	91.5
NBR-PI 2 (DMF)	0.8262	0.9190	0.7869	95.2
NBR-PI 3 (DMF)	0.6315	0.7746	0.5728	90.7
NBR-PI 4 (DMF)	0.5896	0.6375	0.5381	91.3

Figure S4. Digital photographs of the rubber samples before and after gel fraction measurements, along with the corresponding gel fractions (%).

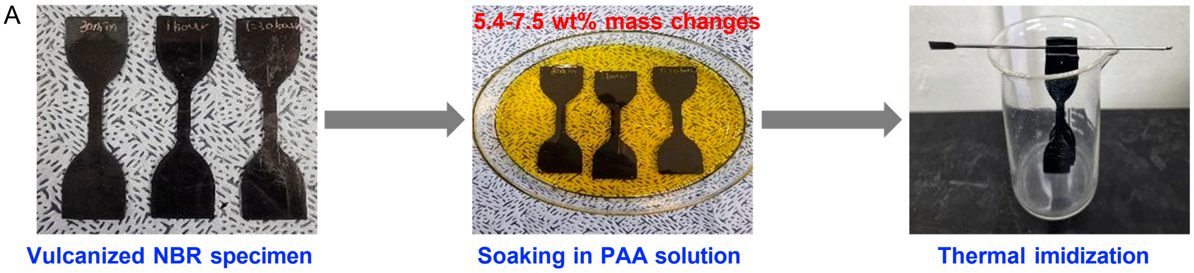

B

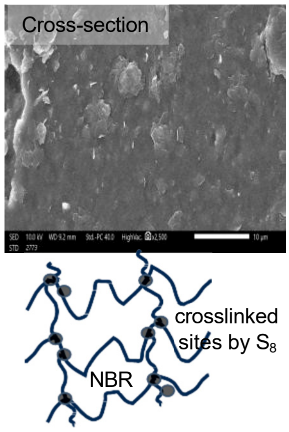

Property	NBR			NBR-PI semi-IPN		
Aging at 100 ℃ for 72 h	Before	After	Change (%)	Before	After	Change (%)
Hardness (Shore A)	85	88	3.5	85	92	8.2
Tensile (kgf/cm^2)	15.4	17.8	16.0	12.5	14.3	14.1
Elongation (%)	216.3	121.4	43.8	189.5	93.4	50.7
100% modulus (MPa)	9.1	16.3	78.8	9.4	13.3	41.5

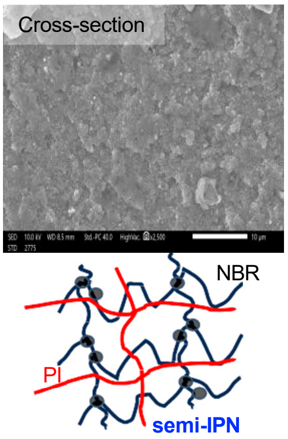

Figure S5. Conventional preparation of NBR-PI semi-IPNs via a modified soaking method (A), along with cross-sectional images and mechanical properties of vulcanized NBR and NBR-PI semi-IPNs (B).
